# Treatment of renal lower pole stones: an update

**DOI:** 10.1590/S1677-5538.IBJU.2020.1023

**Published:** 2021-01-20

**Authors:** Eduardo Mazzucchi, Fernanda C.G. Berto, John Denstedt, Alexandre Danilovic, Carlos Alfredo Batagello, Fabio C.M. Torricelli, Fabio C. Vicentini, Giovanni S. Marchini, Miguel Srougi, William C. Nahas

**Affiliations:** 1 Faculdade de Medicina da Universidade de São Paulo Hospital das Clínicas Divisão de Urologia SP Brasil Seção de Endourologia-Divisão de Urologia, Hospital das Clínicas, Faculdade de Medicina da Universidade de São Paulo, SP, Brasil;; 2 Western University Ontario Division of Urology Canada Division of Urology, Western University Ontario, Canada

## INTRODUCTION

The prevalence of urinary lithiasis ranges from 8% to 19% in males and 3% to 5% in females in Western countries and varies greatly worldwide (1). These numbers are rising particularly due to changes in lifestyle including a higher intake of animal protein and carbohydrates and less physical activity, leading to an increased prevalence of obesity and diabetes (1-3). These changes also reflect in the gender gap and stone composition (4). Clinical symptoms, mainly renal pain, develop in approximately 50% of urinary stone patients and require intervention (5). Additionally, 50% of the affected patients will experience a recurrence during their lifetime (6). Lower pole stones (LPS) account for approximately 35% of renal calculi and may remain asymptomatic in many patients. On the other hand, treatment of such stones is challenging due to the difficulty in eliminating fragments and anatomical access to the inferior renal calyx (7). A great debate has arisen regarding the best management of LPS and many controversies still exist since large randomized studies are scarce in the literature. Some reviews and metanalysis have been published and made a significant contribution for a better understanding of this issue but, unfortunately, many of these reviews are based in heterogeneous and low-quality studies. Currently, the management of lower pole stones includes watchful waiting, extracorporeal lithotripsy (SWL), flexible ureterorenoscopy (FURS) and percutaneous nephrolithotripsy (PCNL) with its variations including mini, ultramini, micro and supermini PCNL. The success rates for each of these treatment modalities is related to stone burden and composition, patient’s body habitus and anatomy as well as the surgeon’s experience. The main differences among these modalities is related to their different degrees of invasiveness, anesthetic requirement, stone clearance, complications and costs (8).

The aim of this article is to review the results of each of these treatment modalities according to the stone burden and patient’s characteristics in order to help urologists decide what is the optimal approach to manage lower pole stones in each patient.

## MATERIALS AND METHODS

**Figure 1:**
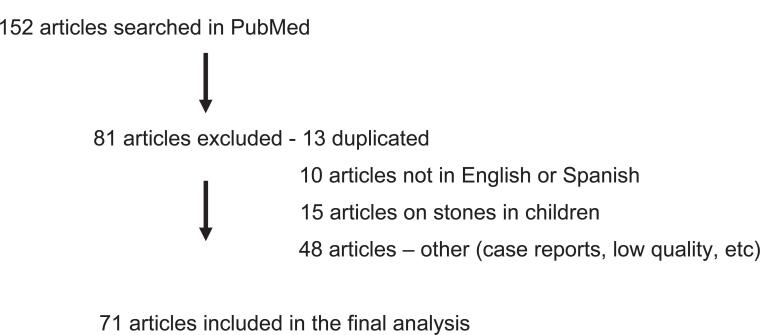
Flowchart of the study.

A PubMed database search was conducted in August-September 2020 using the following Medical Subject Heading (MeSH) terms in several combinations: lower pole, lower calyx, inferior calyx, renal stones, urinary stones, urinary lithiasis, renal anatomy, treatment, extracorporeal lithotripsy, flexible ureteroscopy, percutaneous nephrolithotripsy, costs. We included original articles published between 1990 and 2020, in English or Spanish languages. Studies involving children, review articles, and case reports were not included. Initially 152 articles were reviewed, 81 studies were not analyzed due to reasons shown in the flowchart, 71 articles were included in the final analysis. The flowchart of the study is shown in Figure-1.

We aimed to present, in a summarized way, the optimal treatment for lower pole calculi according to the stone burden, taking into consideration the stone-free rates, hospitalization and complications of each type of procedure.

### Renal anatomy-The inferior calyx

The anatomy of the collecting system may influence the treatment outcome for kidney stones (9). Sampaio and Aragão, in 1992, described the lower pole spatial calyceal anatomy using an endocast and suggested that some anatomic features could impact fragment clearance (10). Sampaio et al. showed that the angle between the calyx where the stone is located and the renal pelvis (infundibulopelvic angle) influences stone elimination (11). Among 146 endocasts obtained from 73 adult cadavers, 74% presented an obtuse infundibulopelvic angle in the lower pole, 60.3% had lower pole infundibula with diameters ≥4mm and 56.8% of the lower poles drained multiple calyces (12). In a study published in 1997 (11), the authors described a technique to measure the infundibulopelvic angle. The first line (line I) links the central axis of the superior ureter with the central axis of the ureteropelvic junction and the second line (line II) follows the orientation of the main infundibulum in the lower calyx where the stone is located. The infundibulopelvic angle is measured between lines I and II. In another study with 74 patients submitted to SWL for LPS, 52 presented an infundibulopelvic angle >90° (obtuse) and 75% of them eliminated the fragments within 3 months while in patients with an infundibulopelvic angle <90° (acute) the clearance rate was 23% (12). Although Elbahnasy et al. (13) used a slightly different method to measure the infundibulopelvic angle, in their cohort with 34 patients, significant larger infundibulopelvic angles were identified in stone-free patients following SWL (75 vs. 51 degrees, p=0.009). A retrospective study with 116 patients comparing five different anatomic characteristics, demonstrated that infundibulopelvic angle was the only significant factor to predict stone-free rates after SWL (34% in patients with an acute angle vs. 66% for obtuse angle, p=0.012) (14).

The infundibular length, measured from the bottom of the infundibulum to the lower lip of the renal pelvis (13), is also related to stone clearance. In a study with 13 patients who underwent ureteroscopy and 21 submitted to SWL, shorter infundibular lengths (≤3cm) were related to better stone clearance following SWL (13). Another study with 151 patients comparing SWL, PCNL and FURS also demonstrated higher SFR in patients submitted to SWL with an infundibular length <3cm (15).

Narrow infundibula (<4mm) (10) and complex lower pole calyceal anatomy are also related to lower SFR in several studies (1, 11, 13, 16) although another study with a large number of patients did not demonstrate significant difference in SFR when analyzing these specific characteristics (14). The presence of two or more favorable or unfavorable characteristics, though, was more significant than any isolate factor (15).

An acute infundibulopelvic angle is related to a decreased stone clearance following treatment. Longer lower pole infundibulum length with narrow diameters and complex calyceal anatomy seem to be related to lower SFR in LPS.

### General overview of the current available methods of LPS treatment

SWL is a non-invasive method of stone treatment introduced in 1980, performed under sedation and in an outpatient setting with a low complication rate (17). The success of SWL is highly affected by:

- Stone burden-the likelihood of success decreases for stones >20mm.
- Stone composition-cystine, brushite and very soft stones (struvite) with a high amount of organic matrix (18).

When stone composition is unknown, stone density (as measured by Hounsfield units on CT) can predict stone fragility and response to SWL.

According to Joseph et al., the stone-free rate (SFR) for stones with less than 500HU, between 500-1000HU and more than 1000HU is 100%, 87.5% and 54.5%, respectively (19).

- Stone location-LPS represent a challenge to SWL especially when the infundibulopelvic angle is acute; results are poorer when compared to stones located in the upper pole or renal pelvis. In the multicentric trial study Lower Pole I, conducted by Albala et al., postoperatively SFR at 3 months for LPS treated with SWL in comparison to PCNL was 95% for PCNL versus 37% for SWL (20).
- Obesity-obese patients represent a challenge for SWL, first because there are weight limitations on the lithotripter table or gantry, the second challenge is positioning patients with high body mass index (BMI) to target the stone as the focal length of most lithotripters varies from 12-16cm (18), and third, the excess adipose tissue dampens energy from the shock wave as it travels to the focal point (21).

FURS is a minimally invasive method that gained popularity among urologists due to its high success rates and low incidence of complications. The method is not affected by obesity and can be performed in anticoagulated patients or during pregnancy, under general or spinal anesthesia and in outpatient or short hospitalization settings. The major drawback of FURS is its costs; many disposables such as guide wires, baskets, access sheaths and laser fibers might be needed in a regular procedure, although they are not mandatory in all situations.

PCNL is the most efficient method in terms of stone-free rates but is also the one with the highest morbidity among all the above cited methods, and the complications include bleeding, lesion of adjacent organs and urinary extravasation. PCNL has passed through changes in recent years especially regarding miniaturization of the tracts and new sources of energy aiming the reduction of complications while maintaining the same SFR. Currently, miniperc (16-18/20Fr), ultramini perc (11-14Fr), the microperc (<10Fr) and other variations are described in the literature. Generally, miniaturized PCNL is indicated for stones <30mm. Kirac et al. published a comparison between miniperc and FURS for LPS <15mm and found 91% SFR for both methods with a shorter operative time but longer hospital stay for miniperc (22).

Despite the known advantages and disadvantages of each method, controversies on the treatment of lower pole stones arise and many of them are still not resolved in the literature. Some of the advantages and disadvantages of each method are summarized in Table-1 (23).

**Table 1:** Advantages and Disadvantages of methods for LPS treatment (modified from Moore SL et al.) (23).

Treatment method	Advantages	Disadvantages
SWL	Non-invasive; Outpatient setting; Performed under sedation;Low incidence of complications; Severe complications are rare	Highly dependent on stone burden; Low efficiency for stones > 900HU and obese patients (skin-stone distance >10 cm); Low SFR for LPS; Contraindicated for pregnant patients and coagulopathies; High capital equipment cost
FURS	Minimally invasive; Short hospital stay; Suitable for all types of stones; Low incidence of complications; Major complications are rare	Unable to reach lower pole in some cases; Residual fragments; High cost of disposables; Need for postoperative stent
PCNL	High SFR; Short procedural time; (compared to FURS); Good cost-benefit	Invasive; Contra-indicated in coagulopathies; Higher incidence of complications than SWL and FURS; Major complications possible (hemorrhage, colonic lesions, hemothorax); Longer hospitalization

SWL = Extracorporeal shockwave lithotripsy; PCNL = Percutaneous nephrolithotripsy; FURS = Flexible ureteroscopy

### Asymptomatic lower pole stones <10mm-active surveillance

The natural history of asymptomatic renal stones is controversial. Glowacki et al. (24) evaluated the outcome of asymptomatic calyceal stones and estimated the risk of a symptomatic stone episode or need for intervention to be approximately 10% per year with a cumulative 5-year event probability of 48.5%. Inci et al., in a prospective study evaluating the outcome of asymptomatic lower pole stones, reported 33% disease progression (defined as pain experienced during follow-up, stone growth or need for intervention) and 11% intervention rates during a 52-month follow-up period (25). Koh et al. reported overall incidences of spontaneous stone passage, progression and intervention for asymptomatic calyceal stones of 20%, 45.9% and 7.1%, respectively (26). Based on the current literature, the American Urological Association (AUA) recommends watchful waiting as a valid option for asymptomatic lower pole stones (27, 28). The European Association of Urology (EAU) extends this recommendation to patients with lower pole stones up to 15mm (29). The exceptions to these recommendations are patients with a solitary kidney, those with poor access to medical assistance, airline pilots and military personnel. Additionally, treatment should be recommended if urinary infection develops (27, 28).

In patients under active surveillance for asymptomatic stones, imaging studies are highly recommended every six or 12 months in order to assess stone growth, hydrocalyx or the formation of new calculi (30).

### Interventional treatment of lower pole stones <10mm

There are two main treatment modalities for stones <10mm located in the lower pole: SWL and FURS. PCNL is considered a second line treatment.

Extracorporeal shockwave lithotripsy is a good choice for small caliceal stones due to its noninvasive nature. SWL is more acceptable by patients when compared to FURS (31). Results depend greatly on stone characteristics, especially stone burden and density, body habitus and on renal anatomy, including infundibulum-pelvic angle, length and diameter of the infundibulum. Torricelli et al. reported overall fragmentation, success and stone-free rates of 76%, 54% and 37%, respectively in a prospective study with non-contrast CT performed 90 days after the procedure (32). The authors reported better results for patients with BMI <30kg/m2, stones ≤10mm, stone density <900HU and an infundibular length of 25mm or less. Hoag et al. reported a success rate of 57% (residual fragments less than 3mm) after SWL for LPS <10mm (33). Similarly, in the 3-month follow-up, Pearle et al. reported a stone-free rate of 35% for a single-session SWL and 50% for FURS. The operative time was shorter for SWL when compared to FURS (66 minutes x 90 minutes). Minor intra and post-operative complications were 25% for SWL and 40% for FURS. Patients submitted to SWL reported better quality of life when compared to FURS (31). The influence of lower pole anatomy on fragment clearance and better SFR after SWL is controversial: Elbhanasy et al., reported in 1998 that a wide infundibulopelvic angle or short infundibular length and broad infundibular width are significant favorable factors for stone clearance following ESWL (13). Sener et al. reported 91.5% success (residual fragments <3mm) for SWL and 100% for FURS in a prospective study where results were checked with KUB+ultrasound performed three months after procedure, but patients treated by SWL needed a mean of 2.7 procedures to achieve these results (34). The stone-free rate for FURS reported by Orywall et al. was 89% in the first post-operative day, evaluation was performed by KUB+ultrasound (35), similar to the results obtained by Jessen and colleagues with a SFR of 88.3% in 111 patients with a total mean stone size of 7.47±3.95mm (36), and Perlmutter et al. who reported a SFR of 90.9% in 44 patients with LPS of median 6.89mm (37).

### Treatment of lower pole stones 10-20mm

Patients with medium sized LPS, between 10 and 20mm, can be treated with SWL, FURS and PCNL, especially in its variants including miniperc, ultraminiperc, super mini PCNL, and microperc. Miniperc refers to all percutaneous surgeries performed with a tract that has a diameter smaller than 20Fr. This way, there are variants like mini-perc (16-20Fr), ultra-mini perc (12-14Fr), super-mini perc (12Fr) and micro-perc (4.8Fr-All-seeing-needle^®^). There are no studies comparing these modalities in the treatment of LPS.

SWL stone-free rates are more influenced by stone burden and composition and by patient’s body habitus, overall obesity as well as renal anatomy as previously stated (38). Many studies comparing the results of SWL, FURS and PCNL have been published and some of them are summarized in Table-2 (39-47).

**Table 2:** Stone-free rates for LPS 10-20 mm at two or three months after treatment.

	Type of study	N° of patients	SWL (%)	FURS (%)	Miniperc (%)	p-value
Bozkurt et al., 2011 (39)	Retrospective	79	-	89.2	92.8	0.571
El Nahas et al., 2012 (40)	Retrospective	89	67.7	86.5	-	0.038
Kumar et al., 2015 (41)	Prospective	180	78.4	85.4	-	0,34
Vilches et al., 2015 (42)	Prospective		41.2	75.0	-	<0.05
Kumar et al., 2015 (43)	Prospective	126	73.8	86.1	95.1	0.01
Chan et al., 2017 (44)	Retrospective	225	48.5	42.9	66.7	0,59
Zeng et al., 2018 (45)	Prospective	160	-	82.5	93.8	0.028
Ozgor et al., 2018 (46)	Retrospective	241	77.9	89.0	-	0.029
Jin et al., 2019 (47)*	Prospective	220	-	97.3	99.1	0.622

* stone free considered as the occurrence of fragments <3mm

Stone-free rates, which means the complete absence of residual fragments, are higher for patients submitted to PCNL (and its variations) and FURS when compared to SWL in most of the studies. Regarding micro-perc, the reported stone-free rate is 85% but there is only one retrospective article published on this modality of treatment for lower-pole stones (48).

The re-treatment rate (63.4% vs. 2.1% and 2.2%, p <0.001) and the need for auxiliary procedures (20.2% vs. 8.8% and 6.6%, p <0.02) are significantly higher for patients submitted to SWL when compared to those treated with FURS or miniperc (43). The mean number of SWL sessions required to reach good results is 2.8 (46).

Operative time ranges from 26 to 49 minutes for SWL, 39 to 52 minutes for FURS and 60 minutes for PCNL with no significant differences among the three modalities (41). Although in a study including stones in different renal locations, supine miniperc presented shorter operative and fluoroscopy times compared to procedures performed in prone position (58.1±45.9 vs. 80.1±40.0 min, p=0.025 and 3.0±1.7 vs. 4.9±4.5 min, p=0.01, respectively) (49).

Mean hospital stay ranged between 2.1 and 3.1 hours for SWL, 21.1 and 31.1 hours for FURS and 74.4 hours for miniperc (43, 50).

One of the advantages of FURS, is the possibility of treating patients submitted to anticoagulation therapy and during pregnancy. Also, there is no influence of obesity on its results. For stones with >15mm, though, fatigue could impact in the results and Hui et al. suggests that this can be minimized by a two-shift operation which also would increase the clearance rates (51).

Complications of these procedures are, in general, minor, and are classified as grades 1, 2 or 3 (6%, 12% and 5% respectively) by the Clavien-Dindo modified classification (52). The most common complications are pain, gross hematuria, fever, urinary tract infection and sepsis. Urinary tract infection and gross hematuria are more frequent in patients submitted to mini PCNL (43) and pain is more frequent in those treated by SWL (41). Steinstrasse after SWL is reported to occur in 4% of patients with stones less than 20mm, rising to 5-10% for stones >20mm (53). Sepsis is the most serious complication and occurs between 0.9% and 5% of the cases (47). Severe complications like ureteral avulsion, arteriovenous fistulae and severe kidney injuries have been described after FURS but are rare (54). Complications are higher for miniperc when compared to SWL and FURS but not significantly (43, 44).

Transfusion is rarely needed in patients submitted to SWL or FURS, but for patients treated by miniperc, the rate is around 4% (50). Mortality is low in FURS and six cases have been described in the literature until 2016, four of them due to sepsis, one due to hemorrhagic complications and another one because of anesthetic complications. In three of the four cases of sepsis, non-treated UTI was present before surgery (55). The mortality rate among patients submitted to PCNL is reported to be 0.03% generally due to sepsis and hemorrhage (56).

In the author’s view, choosing between FURS and miniperc for the treatment of 10-20mm LPS will depend on several factors including stone burden, patient’s body habitus, clinical condition and expectations, type of equipment the surgeon has access to (laser, scopes, fibers, etc.) and his expertise in one or the other method.

### Repositioning of LPS

The technique of lower calyx displacement to an upper or medium calyx where the access is easier was first described by Kourambas and colleagues (57) and more details were discussed by Auge et al. in 2001 (58). The aim of this maneuver is to improve stone-free rate and reduce ureteroscope damage. Schuster et al. reported an improvement in the success rate for LPS 10-20mm from 29% to 100% in cases where repositioning was used (59). There are still controversies on this issue once the literature is scarce with small numbers of studied cases, but a recent survey showed that 56% of urologists reposition lower pole stones during flexible ureteroscopy (60).

### Influence of scope type on results of FURS for lower-pole stones

With the advent of digital flexible ureteroscopes and, more recently, single-use digital scopes, a question arises: do these new devices improve results and reduce complications of lower-pole stone treatment? One study compared prospectively the Polyscope^®^, a first generation single-use flexible ureteroscope with the Olympus P5^®^, a re-usable optical flexible scope. The single session SFR, for lower calyceal stones, was significantly better for URF-P5^®^than Polyscope^®^ (82.0% vs. 69.2%, p=0.022) and the complication rate presented no difference (15.3% vs. 15%, p=0.3) (61). A more recent study compared the Olympus P6^®^, a last generation re-usable fiberoptic scope with the Lithovue^®^, a first-generation single-use digital scope and reported a lower complication rate for the single-use scope (5.4% vs. 18.0%, p <0.05) (62). In another study, not addressed for LPS only, but for stones located in all calyces, there was no difference in SFR and complications when comparing the Lithovue^®^ with the Storz Flex X2S^®^ (last generation fiberoptic) and the Flex XC^®^ (digital) (63).

Although a definite study proving the advantage of one type of flexible ureteroscope for treatment of stones located in the lower pole still does not exist, studies have been published showing that there is a correlation between the technical difficulty of the procedure, like treating lower-pole stones with a very acute infundibulo-pelvic angle, and a higher incidence of ureteroscope malfunction (64). The combination of aggressive active deflection of the flexible ureteroscope and simultaneous passage of the holmium laser probe may stress the fiberoptic system and result in fiber breakage (65). Ozimek et al. evaluated the occurrence of damage in reusable flexible ureterorenoscopes and found that in 32 of 423 (7.5%) cases the scopes were defective after the procedures. Thirty-one of 32 cases (96.86%) with proven scope damage were related to exploration of the lower pole and in 20 of 23 cases (86.96%) it was for stone treatment in that location (66).

### Treatment of lower pole stones >20mm

Traditionally PCNL has been the most effective method for stones larger than 2cm. According to Pardalidis et al., a 98% SFR was observed after a single session treatment in 48 patients with LPS >2cm, using the conventional 26Fr rigid nephroscope. The mean hospital stay was 2.3 days. Fever was the most common complication, occurring in 6.9% of patients and no cases of hemorrhage were reported (67). New technologies, such as 3D printing have been recently described to facilitate the percutaneous access and reduce operative time, complications (68), and fluoroscopy time (69). Microperc, a miniaturized version of the PCNL which uses a 4.8Fr “All-seeing needle^®^” coupled to an 8Fr microsheath and a 360 micron fiber laser, has been used by some authors for treating LPS up to 29mm with a 85% SFR in the post-operative day30 CT scan (48). Flexible ureteroscopy has been used as an alternative method for treating LPS >2cm. A SFR of 93.3% was reported in a study with 15 patients and stones 20-25mm not only located in the lower calyx. However, the mean number of sessions needed to reach these results was 2.3, ranging from two to four (70). In another study, Takazawa et al. reported a 100% SFR (considering residual fragments <4mm) after a mean of 1.4 sessions per patient (range 2-4). No intra-operative complications occurred and fever was the only complication observed in three of the 20 studied patients (15%) (71). SWL is not recommended for treatment of lower pole stones >2cm according to the AUA and EAU guidelines (27-29).

## CONCLUSIONS

Watchful waiting is recommended for asymptomatic LPS <10mm, except in cases of solitary kidneys and in other particular situations. SWL and FURS are both good options for symptomatic LPS <10mm, FURS has advantages in obese, pregnant and anticoagulated patients. For treating LPS 10 to 20mm, FURS has currently the best cost-benefit, but Miniperc has a higher SFR although with a higher morbidity. Repositioning LPS to a more favorable calyx probably improve results and decreases endoscope damage but more studies are needed. Single-use ureteroscopes are probably recommended for LPS located in calices with a very acute infundibulum-pelvic angle where the chance of endoscope damage is higher. PCNL is the best treatment modality for stones >20mm.
